# Community-Associated Outbreak of COVID-19 in a Correctional Facility — Utah, September 2020–January 2021

**DOI:** 10.15585/mmwr.mm7013a2

**Published:** 2021-04-02

**Authors:** Nathaniel M. Lewis, Amelia Prebish Salmanson, Andrea Price, Ilene Risk, Colleen Guymon, Marcus Wisner, Kyle Gardner, Rena Fukunaga, Amee Schwitters, Lauren Lambert, Henry C. Baggett, Raimi Ewetola, Angela C. Dunn

**Affiliations:** ^1^Epidemic Intelligence Service, CDC; ^2^Utah Department of Health; ^3^CDC COVID-19 Response Team; ^4^Salt Lake County Health Department, Utah; ^5^Utah Department of Corrections, Draper, Utah.

Transmission of SARS-CoV-2, the virus that causes COVID-19, is common in congregate settings such as correctional and detention facilities (*1*–*3*). On September 17, 2020, a Utah correctional facility (facility A) received a report of laboratory-confirmed SARS-CoV-2 infection in a dental health care provider (DHCP) who had treated incarcerated persons at facility A on September 14, 2020 while asymptomatic. On September 21, 2020, the roommate of an incarcerated person who had received dental treatment experienced COVID-19–compatible symptoms*; both were housed in block 1 of facility A (one of 16 occupied blocks across eight residential units). Two days later, the roommate received a positive SARS-CoV-2 test result, becoming the first person with a known-associated case of COVID-19 at facility A. During September 23–24, 2020, screening of 10 incarcerated persons who had received treatment from the DHCP identified another two persons with COVID-19, prompting isolation of all three patients in an unoccupied block at the facility. Within block 1, group activities were stopped to limit interaction among staff members and incarcerated persons and prevent further spread. During September 14–24, 2020, six facility A staff members, one of whom had previous close contact^†^ with one of the patients, also reported symptoms. On September 27, 2020, an outbreak was confirmed after specimens from all remaining incarcerated persons in block 1 were tested; an additional 46 cases of COVID-19 were identified, which were reported to the Salt Lake County Health Department and the Utah Department of Health. On September 30, 2020, CDC, in collaboration with both health departments and the correctional facility, initiated an investigation to identify factors associated with the outbreak and implement control measures. As of January 31, 2021, a total of 1,368 cases among 2,632 incarcerated persons (attack rate = 52%) and 88 cases among 550 staff members (attack rate = 16%) were reported in facility A. Among 33 hospitalized incarcerated persons, 11 died. Quarantine and monitoring of potentially exposed persons and implementation of available prevention measures, including vaccination, are important in preventing introduction and spread of SARS-CoV-2 in correctional facilities and other congregate settings (*4*).

In Utah, the 7-day average daily incidence of confirmed^§^ COVID-19 cases increased from 12 cases per 100,000 population^¶^ on September 1, 2020 to a peak of 106 on November 22, 2020.** On March 6, 2020, facility A had implemented symptom and temperature screening at entry for all staff members and SARS-CoV-2 testing at intake for incarcerated persons. Staff members were required to wear a surgical mask or cloth face covering at work; incarcerated persons were issued cloth face coverings and directed to always wear them. On March 27, 2020, personal protective equipment (PPE) stations were installed, and dedicated nursing staff members were placed on call to supervise PPE use, mostly during intake processing. On May 1, 2020, nonessential visits were stopped.

Before September 14, 2020, no known COVID-19 cases had been diagnosed among incarcerated persons at facility A other than 15 cases among incarcerated persons screened at intake and identified by reverse transcription–polymerase chain reaction (RT-PCR) testing while isolated. On September 14, 2020, a visiting DHCP treated 10 incarcerated persons in a dental clinic at facility A (Table 1). At entry screening, the DHCP had a normal temperature and reported no COVID-19–compatible symptoms but experienced symptoms later that evening. On September 15, 2020, the DHCP received SARS-CoV-2 RT-PCR testing and notified the facility of a positive result 2 days later (September 17, 2020). The DHCP was classified as patient DHCP1 (the index patient). By September 24, 2020, COVID-19 was confirmed in three incarcerated persons, and the outbreak subsequently expanded to include 198 incarcerated persons and seven staff members by October 3, 2020.

**TABLE 1:** Clinical and exposure characteristics of incarcerated persons (IPs) with COVID-19 (n = 9), a visiting dental health care provider (DHCP1),* and potentially infectious staff members who worked near block 1^†^ areas or patients in correctional facility A — Utah, September 14–September 26, 2020

Patient no. (occupation)	Preexisting conditions and risk factors	Date of symptom onset^§^	Symptoms reported	Date of positive RT-PCR test result^¶^	Known exposure (duration)**	Location of potential onward facility exposures^††^
**Visiting staff member case**						
DHCP1/S1	Unknown	Sep 14, 2020	Chills, muscle aches, fatigue	Sep 15, 2020	Community contact (unknown)	Dental clinic
**IP resident cases associated with nonfacility (visiting) health care provider**						
R1 (IP)	Emphysema, history of smoking	Sep 21, 2020	Chills, muscle aches, runny nose, sore throat, cough, headache, fatigue	Sep 23, 2020	Contact to R2 (ongoing)	Block 1
R2 (IP)	Depression, history of smoking	Unknown	Headache	Sep 23, 2020	Contact to S1: surgical tooth extraction (15 mins); roommate of R1	Block 1
R3 (IP)	Asthma, lipidemia, developmental disabilities	Unknown	None	Sep 24, 2020	Contact to S1: biopsy and evaluation (12 mins)	Block 1
**Staff member cases with known close contact with block 1 confirmed IP cases**						
S2 (officer)	Chronic gastrointestinal	Sep 23, 2020	Subjective fever, chills, sore throat, cough, fatigue, loss of taste, loss of smell	Sep 24, 2020	Contact to IP (R2) during interview (>15 mins cumulative)	Block 1
**Staff member cases with possible or indirect contact with block 1 IP cases and staff members with COVID-19**						
S3 (maintenance worker)	Type 2 diabetes, cardiovascular disease	Sep 17, 2020	Muscle aches, cough, fatigue	Sep 18, 2020	Community contact (unknown)	Block 2 culinary facility, corridor^†^
S4 (officer)	None	Sep 18, 2020	Subjective fever, chills, muscle aches, headache, fatigue	Sep 21, 2020	Household contact (ongoing)	Block 2 corridor^†^
S5 (maintenance worker)	Unknown	Unknown	Unknown	Sep 20, 2020	Contact to S4 (ongoing)	Block 2
S6 (officer)	None	Sep 21, 2020	Fever, subjective fever, chills, muscle aches, runny nose, sore throat, cough, difficulty breathing, nausea, headache, fatigue, abdominal pain, diarrhea	Sep 23, 2020	Unknown (unknown)	Corridor^†^
S7 (HCP)	None	Sep 24, 2020	Chills, muscle aches, runny nose, sore throat, cough, headache, fatigue, loss of taste, loss of smell	Sep 26, 2020	Household contact with same date of symptom onset (ongoing)	Infirmary

**Abbreviations:** HCP = health care provider; RT-PCR = real time reverse transcription–polymerase chain reaction; R = resident; S = staff member.

* DHCP1 was the first reported staff member with COVID-19; block 1 cases that occurred among incarcerated persons were associated with exposure to this staff member.

^†^ Block 1 is a residential unit with two-person, closed-door rooms where COVID-19 was identified in IPs; block 2 is a residential unit with single-person, open-door rooms, where COVID-19 was next identified in IPs; blocks 1 and 2 are connected by a corridor (60-ft-long, 12-foot-wide) that staff members use occasionally to travel between blocks 1 and 2.

^§^ Date of any COVID-19 symptom first reported.

^¶^ Specimen collection date.

** Where known, exposures involve contact to a confirmed case with an earlier onset date unless otherwise specified.

^¶¶^ Facility locations include dental clinic, block 1, block 2, corridor between blocks 1 and 2, culinary facility serving blocks 1 and 2, and the infirmary.

The outbreak investigation started on September 30, 2020. To better understand factors contributing to the outbreak, investigators interviewed facility dental and medical staff members during September 30–October 9, 2020. Investigators also reviewed case records of staff members who reported onset of COVID-19–compatible symptoms during September 14–September 24, 2020 and who worked in block 1 or in other areas where possible exposure to block 1 incarcerated persons or staff members might have occurred. This activity was reviewed by CDC and was conducted consistent with applicable federal law and CDC policy.^††^

On September 14, 2020, DHCP1 wore a valveless N95 respirator face mask at entry to facility A, during temperature and symptom screening, and in transit to the dental clinic. During treatment, DHCP1 wore the N95 as well as a gown, gloves, and goggles, and changed gowns and gloves after each patient. Among 10 incarcerated persons (residents) who received treatment, six (including a resident who subsequently developed COVID-19 [patient R2]) had surgical tooth extractions (a 15-minute procedure), one (patient R3) had a combined evaluation and biopsy (12-minute procedure); and three had 10-minute patient evaluations. All 10 incarcerated persons were interviewed for 5 minutes each by one of five facility dental clinic staff members, all of whom wore recommended PPE. On the day of treatment, none of the incarcerated persons was tested for SARS-CoV-2, screened for fever or symptoms, or wore masks or gloves during treatment.

On September 21, 2020, patient R1 (the roommate of patient R2, who had received dental treatment) experienced symptoms and visited the infirmary the next day. On September 23, 2020, patients R1 and R2 both received positive SARS-CoV-2 RT-PCR test results; patient R2 was tested because of his close contact with patient R1, despite being asymptomatic at the time (he retrospectively reported a headache with indeterminate onset) (Supplementary Figure, https://stacks.cdc.gov/view/cdc/104506). Patients R1 and R2 were moved from block 1 to an unoccupied isolation block, and staff members began wearing N95 respirators and eye protection. On September 24, 2020 the nine remaining incarcerated persons treated by DHCP1 were tested. Patient R3, who also lived in block 1, received a positive result and was isolated.

On September 25, 2020, facility medical staff members tested specimens from the remaining 171 block 1 incarcerated persons with unknown SARS-CoV-2 infection status (Figure); 46 (26%) received positive RT-PCR test results. The incarcerated persons with positive results were isolated, and the remaining persons living in block 1 were quarantined; those with negative test results and no known exposures were placed together in rooms in block 1, and those with known exposure were quarantined in single-occupant rooms in another unoccupied area. On October 1, 2020, specimens collected from incarcerated persons who lived in block 1 and who had received a negative result on September 25, 2020 were tested; 57 (46%) of 127 received a positive result. Among 174 incarcerated persons living in block 1, a cumulative total of 106 (61%) had received a positive result by October 1, 2020; through January 31, 2021, a total of 117 cases occurred among block 1 incarcerated persons. No hospitalizations or deaths among block 1 cases were reported, but several patients (including patient R2) were treated in the facility infirmary. Facility A medical staff members indicated that symptoms of incarcerated persons were not consistently recorded because of workload constraints as well as patients’ hesitancy to report symptoms to avoid being moved; among 11 patients with data available, six reported symptoms. On October 1, 2020, two additional incarcerated persons with COVID-19–compatible symptoms who lived permanently in a long-term care setting within the infirmary, also received positive SARS-CoV-2 RT-PCR test results.

**FIGURE:**
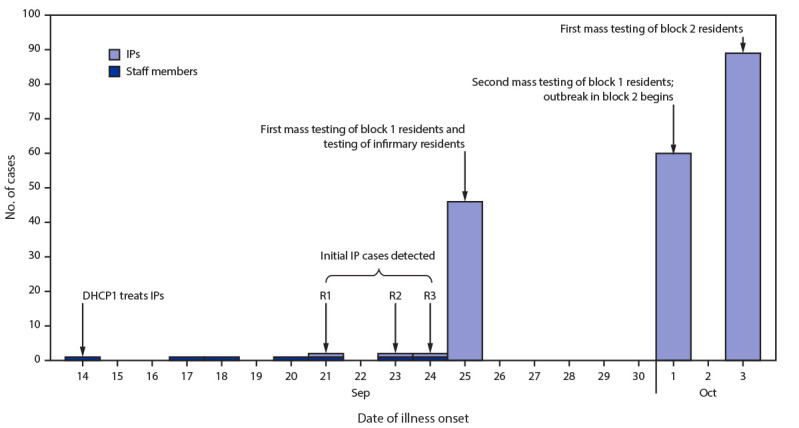
Number of COVID-19 cases (N = 205) among incarcerated persons* (IPs) (n = 198)and staff members^†^ (n = 7) associated with initial outbreak at correctional facility A,^§^ by date of illness onset^¶^ — Utah, September 14–October 3, 2020 **Abbreviations:** DHCP1 = dental health care provider; R = resident. * IPs included R1: confirmed case in a resident IP treated by DHCP1; R2: confirmed case in roommate of patient R1 (resident IP index case); and R3: second confirmed case in IP treated by DHCP1. ^†^ DHCP1 is the first case in a staff member at correctional facility A. ^§^ Block 1 is the first residential unit at correctional facility A where COVID-19 was identified in IPs; block 2 is the second residential unit where COVID-19 was identified in IPs; block 1 and block 2 are connected by a corridor. ^¶^ Where date of illness onset was unknown or when symptoms data were not available, date of specimen collection with first positive test result is used.

Block 2 is connected to block 1 by a corridor (60-ft long, 12-ft wide) through which staff members occasionally travel. Among 174 incarcerated persons in block 2, three with COVID-19–compatible symptoms were moved to the isolation area on October 1, 2020 after receiving positive test results. On October 3, 2020, RT-PCR testing of specimens from the 171 remaining block 2 incarcerated persons identified an additional 89 cases (Figure) (Table 2). Among the 92 (53%) incarcerated persons in block 2 with positive results by October 3, 2020 additional information was available for 20 (22%), including five who were symptomatic, one of whom was hospitalized on October 12, 2020 and died on November 14, 2020. As of October 3, 2020, a total of 198 cases among incarcerated persons had been reported in the facility. By January 31, 2021, the outbreak had spread to six of eight residential units (consisting of 14 of 16 blocks); 1,368 cases had been reported and the median attack rate in all affected blocks, including blocks 1 and 2, was 69% (range = 7%–96%) and the overall attack rate in facility A was 52%. Among blocks with cases, the attack rate was higher in dormitory-style or open-cell blocks (76%) compared with single or paired closed-door cells (64%).

**TABLE 2:** Total* COVID-19 cases, hospitalizations, and deaths among incarcerated persons (IPs) and staff members, blocks 1 and 2^†^ in correctional facility A — Utah, September 14, 2020–January 31, 2021

Case characteristics among IPs and staff members	Facility A	Block 1	Block 2
**IPs**			
**Total no. of residents**	**2,632**	**174**	**174**
No. of COVID-19 cases (% attack rate^§^), initial outbreak^¶^	198 (8)	106 (61)	92 (53)
No. of COVID-19 cases (% attack rate^§^), total	1,368 (52)	117 (67)	165 (95)
No. of hospitalizations (hospitalization rate**)	31 (22.6)	0 (—)	1 (6.1)
No. of deaths (death rate^††^)	11 (6.5)	0 (—)	1 (6.1)
**Staff members**			
**Total no. of staff members**	**550**	**N/A**	**N/A**
No. of COVID-19 cases (% attack rate^§^), initial outbreak^¶^	7 (1)	N/A	N/A
No. COVID-19 cases (% attack rate^§^), total	88 (16)	N/A	N/A
No. of hospitalizations (hospitalization rate**)	0 (—)	0 (—)	0 (—)
No. of deaths (death rate^††^)	0 (—)	0 (—)	0 (—)

**Abbreviation**: N/A = not available

* Estimated total number of residents as of October 1, 2020; daily counts fluctuated based on intake and release of IP and hiring, termination, or leave among staff members. Staff member total counts, case counts, and attack rates for individual blocks were not available because staff members move among blocks.

^†^ Block 1 is a residential area with two-person, closed-door rooms, where COVID-19 was first identified in IPs; block 2 is a residential area in the same residential unit with single-person, open-door rooms, where COVID-19 was next identified in IPs. Blocks 1 and 2 are connected by a 60-foot-long, 12-foot-wide corridor that staff use occasionally to travel between blocks 1 and 2.

^§^ Attack rate is the number of cases as a proportion of the total number of IPs or staff members. After November 24, 2021, facility A used rapid antigen tests to determine cases in emergency situations.

^¶^ The initial outbreak was defined as September 14–October 3, 2021, and consisted of cases detected in IPs during two mass testing days in block 1 (September 25 and October 1), selective testing of two infirmary residents (October 1), one mass testing day in block 2 (October 3), and seven cases in staff members potentially associated with the outbreak.

** Hospitalizations per 1,000 cases.

^††^ Deaths per 1,000 cases.

Investigations of six cases in staff members occurring during the period from the potential introduction of infection into facility A to the detection of COVID-19 cases in the first three incarcerated persons suggested likely acquisition at work for two (Table 1); staff member patient S2 reported close contact with an infected incarcerated person and staff member patient S5 reported ongoing contact with staff member patient S4. Four staff members (patients S3, S4, S6, and S7) reported only community exposures. Epidemiologic data suggest that cases in patients S3–S7 contributed to the block 2 outbreak (Table 1); however, SARS-CoV-2 might have been introduced into block 1 by infected but asymptomatic or untested staff members. These six staff members (S2–S7) stopped reporting to work after receiving positive test results or learning of their exposure to a person with confirmed COVID-19 (Supplementary Figure, https://stacks.cdc.gov/view/cdc/104506). Cumulatively, as of January 31, 2020, 88 (16%) cases among 550 staff members were reported in facility A.

After 46 cases were detected with mass testing in block 1 on September 27, 2020, facility A notified the Salt Lake County Health Department and the Utah Department of Health. A team from both departments, with technical assistance from CDC, implemented twice-weekly calls with the facility to review infection control guidance, including protocols for cohorting, quarantine and isolation of incarcerated persons and repeated mass testing to identify new cases. The entire facility was placed under a quarantine restriction to limit mobility of staff members among residential units. The state mobile testing team supported mass testing of incarcerated persons and staff member testing events. As of March 2, 2021, the outbreak was ongoing; 1,545 cases (1,452 [94%] among incarcerated persons) have been reported, as well as 31 hospitalizations and 12 deaths among incarcerated persons with COVID-19.

## Discussion

SARS-CoV-2 might have been introduced into correctional facility A by DHCP1 or other staff members with community-acquired infection. The detection of 46 cases just 11 days after the first potential introduction by DHCP1 suggests that infection spread quickly. Infection might also have spread through undetected chains of transmission from staff members working in block 1 to other areas, especially because N95 respirators and eye protection were not usually worn before September 23, 2020.

The possibility of transmission from staff members to incarcerated persons at facility A indicates a need for serial testing for both staff members and incarcerated persons (*1*), as well as careful attention to infection control guidance (*5*), including in health care settings (*6*), where dental treatment is provided (*7*), and in correctional settings (*4*). Screening of nonfacility HCPs with rapid antigen tests, testing incarcerated persons 5–7 days after receiving treatment from nonfacility HCPs, or stopping nonemergency procedures requiring nonfacility staff members could all be considered to reduce introduction and transmission of SARS-CoV-2.

Ten incarcerated persons were exposed to the index patient (DHCP1) on the date of the index patient’s symptom onset and were not immediately quarantined or isolated; two of 10 appeared to be infected by DHCP1. The interval between patient R2’s exposure to R1 (his roommate) and R1’s symptom onset (September 14–21, 2020), suggests a mean 3.5-day incubation for these cases, consistent with previous estimates (*8*). Although only surgical tooth extractions resulted in 15-minute (the longest) exposures to DHCP1, other procedures that require manipulation or prolonged close contact with a patient’s eyes, nose, or mouth might pose a higher risk for transmission during a shorter time frame (*4*). A COVID-19 outbreak among nursing home residents after receiving dental treatment was also reported in New York (*9*).

The findings in this report are subject to at least four limitations. First, it was not possible to determine whether the N95 mask worn by the index patient was fit-tested or working properly, or whether transmission occurred by touching patients’ mucous membranes with contaminated gloves. Poor fit of an N95 respirator can limit its efficacy in preventing the wearer from acquiring or spreading infection (*10*). Second, given the increasing community transmission of SARS-CoV-2 when the outbreak began, SARS-CoV-2 might have been introduced undetected from another essential service provider. Third, inconsistent monitoring and reporting of symptoms could have affected the order in which cases among incarcerated persons were detected. Finally, because whole genome sequencing was not performed, linkages between infections were not ascertained definitively.

Patients exposed to HCPs who are found to be infected with SARS-CoV-2 should quarantine after exposure and be monitored closely (*4*). Because SARS-CoV-2 can spread quickly in correctional and detention facilities (*1*–*3*), particularly in areas with elevated community transmission, control measures are needed to prevent introductions (*4*). Control measures could include regular testing of staff members, rapid testing at entry for visiting HCP, and halting of nonemergency medical procedures requiring outside staff members, as well as universal masking, maintaining physical distancing when possible, and paying attention to hand hygiene. Vaccination of incarcerated persons might help prevent or limit the spread of infection in these facilities.

**Summary**

**What is already known about this topic?**

SARS-CoV-2 transmission is common in congregate settings including correctional and detention facilities.

**What is added by this report?**

Incarcerated persons in a Utah correctional facility were likely exposed to SARS-COV-2 by community-associated sources of introduction, including a visiting dental health care provider. An outbreak in the facility was first detected in the residential block where two residents received treatment; the outbreak spread rapidly, eventually affecting 1,368 (52%) of 2,632 residents (with 31 hospitalizations and 12 deaths) and an estimated 88 (16%) of 550 staff members.

**What are the implications for public health practice?**

Quarantining and monitoring potentially exposed persons are important in preventing the introduction and spread of SARS-CoV-2 infection in correctional facilities and other congregate settings. Vaccination of incarcerated persons might help prevent or limit the spread of infection in these facilities.

## Footnotes

* Includes fever or chill, cough, shortness of breath or difficulty breathing, fatigue, muscle or body aches, headache, new loss of taste or smell, sore throat, congestion or runny nose, nausea or vomiting, or diarrhea.

† Close contact was defined as being within 6 ft of a person with COVID-19 for at least 15 cumulative minutes.

§ For this investigation, a confirmed case was defined as receipt of a positive SARS-CoV-2 real time RT-PCR test result.

¶ Estimate based on an average case count during the previous 7 days per 100,000 population.

** The 7-day cumulative number of new COVID-19 cases in Salt Lake County was obtained from the Utah Department of Health and the Salt Lake County Health Department.

†† 45 C.F.R. part 46.102(l)(2), 21 C.F.R. part 56; 42 U.S.C. Sect. 241(d); 5 U.S.C. Sect. 552a; 44 U.S.C. Sect. 3501 et seq.

## References

[1] Wallace M, Hagan L, Curran KG, COVID-19 in correctional and detention facilities—United States, February–April 2020. MMWR Morb Mortal Wkly Rep 2020;69:587–90. 10.15585/mmwr.mm6919e1 32407300

[2] Hagan LM, Williams SP, Spaulding AC, Mass testing for SARS-CoV-2 in 16 prisons and jails—six jurisdictions, United States, April–May 2020. MMWR Morb Mortal Wkly Rep 2020;69:1139–43. 10.15585/mmwr.mm6933a3 32817597 PMC7439979

[3] Wallace M, Marlow M, Simonson S, Public health response to COVID-19 cases in correctional and detention facilities—Louisiana, March–April 2020. MMWR Morb Mortal Wkly Rep 2020;69:594–8. 10.15585/mmwr.mm6919e3 32407301

[4] CDC. Interim guidance on management of coronavirus disease 2019 (COVID-19) in correctional and detention facilities. Atlanta, GA: US Department of Health and Human Services, CDC; 2021. Accessed December 6, 2020. https://www.cdc.gov/coronavirus/2019-ncov/community/correction-detention/guidance-correctional-detention.html

[5] CDC. Clinical questions about COVID-19: questions and answers. Infection control. Atlanta, GA: US Department of Health and Human Services, CDC; 2021. Accessed November 20, 2020. https://www.cdc.gov/coronavirus/2019-ncov/hcp/faq.html#Infection-Control

[6] CDC. Interim U.S. guidance for risk assessment and work restrictions for healthcare personnel with potential exposure to COVID-19. Atlanta, GA: US Department of Health and Human Services, CDC; 2021. Accessed November 14, 2020. https://www.cdc.gov/coronavirus/2019-ncov/hcp/guidance-risk-assesment-hcp.html

[7] CDC. Guidance for dental settings. Atlanta, GA: US Department of Health and Human Services, CDC; 2020. Accessed November 15, 2020. https://www.cdc.gov/coronavirus/2019-ncov/hcp/dental-settings.html

[8] CDC. Interim clinical guidance for management of patients with confirmed coronavirus disease (COVID-19). Atlanta, GA: US Department of Health and Human Services, CDC; 2019. Accessed December 10, 2020. https://www.cdc.gov/coronavirus/2019-ncov/hcp/clinical-guidance-management-patients.html

[9] Nelson P. Schenectady County nursing home outbreak tied to dental hygienist. The Times Union, December 3, 2020. Accessed February 15, 2021. https://www.timesunion.com/news/article/Schenectady-County-nursing-home-outbreak-tied-to-15772220.php

[10] Cichowicz JK, Casey M, D’Alessandro, MM. Respiratory protection vs. source control–what’s the difference? NIOSH Science Blog. Atlanta, GA: US Department of Health and Human Services, CDC; 2020. https://blogs.cdc.gov/niosh-science-blog/2020/09/08/source-control/

