# *Erratum:* Vol. 70, No. 13

**DOI:** 10.15585/mmwr.mm7015a5

**Published:** 2021-04-16

In the report “Community-Associated Outbreak of COVID-19 in a Correctional Facility — Utah, September 2020–January 2021,” on page 467 in the first paragraph, the fourth sentence should have read, “Two days later, the roommate received a positive SARS-CoV-2 test result, becoming the first person with a **known community-associated** case of COVID-19 at facility A.”

